# Progesterone Receptor Membrane Component 1 Mediates Progesterone-Induced Suppression of Oocyte Meiotic Prophase I and Primordial Folliculogenesis

**DOI:** 10.1038/srep36869

**Published:** 2016-11-16

**Authors:** Meng Guo, Cheng Zhang, Yan Wang, Lizhao Feng, Zhengpin Wang, Wanbo Niu, Xiaoyan Du, Wang Tang, Yuna Li, Chao Wang, Zhenwen Chen

**Affiliations:** 1 Department of Laboratory Animal Science, School of Basic Medical Science, Capital Medical University, Beijing 100069, Peoples’ Republic of China; 2 College of Life Science, Capital Normal University, Beijing 100048, People’s Republic of China; 3 Department of Medical Genetics and Developmental Biology, School of Basic Medical Science, Capital Medical University, Beijing 100069, Peoples’ Republic of China; 4 State Key Laboratory for Agro-Biotechnology, College of Biological Science, China Agricultural University, Beijing 100193, Peoples’ Republic of China; 5 Laboratory of Cellular and Development Biology, NIDDK, National Institutes of Health, Bethesda MD 20892, USA

## Abstract

Well-timed progression of primordial folliculogenesis is essential for mammalian female fertility. Progesterone (P4) inhibits primordial follicle formation under physiological conditions; however, P4 receptor that mediates this effect and its underlying mechanisms are unclear. In this study, we used an *in vitro* organ culture system to show that progesterone receptor membrane component 1 (PGRMC1) mediated P4-induced inhibition of oocyte meiotic prophase I and primordial follicle formation. We found that membrane-impermeable BSA-conjugated P4 inhibited primordial follicle formation similar to that by P4. Interestingly, PGRMC1 and its partner serpine1 mRNA-binding protein 1 were highly expressed in oocytes in perinatal ovaries. Inhibition or RNA interference of PGRMC1 abolished the suppressive effect of P4 on follicle formation. Furthermore, P4-PGRMC1 interaction blocked oocyte meiotic progression and decreased intra-oocyte cyclic AMP (cAMP) levels in perinatal ovaries. cAMP analog dibutyryl cAMP reversed P4–PGRMC1 interaction-induced inhibition of meiotic progression and follicle formation. Thus, our results indicated that PGRMC1 mediated P4-induced suppression of oocyte meiotic progression and primordial folliculogenesis by decreasing intra-oocyte cAMP levels.

In mammalian females, the pool of primordial follicles established by primordial folliculogenesis represents nearly all the germ cells available throughout their reproductive life^1^. In mice, primordial germ cells enter genital ridge and divide mitotically, with incomplete cytokinesis. Consequently, clusters of germ cells, which are linked by cytoplasmic bridges, form germline cysts^2^. Beginning at 13.5 days post coitum (dpc), the germ cells enter meiosis and pass through leptotene, zygotene, pachytene, diplotene, and dictyate stages of meiotic prophase I^3^,^4^. Accompanied by meiotic progression, cysts begin to break apart and two-third oocytes undergo apoptosis at 17.5 dpc^5^. After birth, the surviving oocytes successively arrest in the dictyate stage, which is indispensable to folliculogenesis^6^,^7^. Then nearly all primordial follicles are established within 1–3 days post parturition (dpp). Although appropriate folliculogenesis is vital for female fertility, mechanisms underlying this process remain to be determined.

Several recent studies, including our previous study, showed that progesterone (P4) is critical for regulating primordial follicle formation in several mammalian species. In murine perinatal ovaries, precipitous decline of high P4 levels and assembly of abundant primordial follicles occur simultaneously after labor^8^,^9^,^10^. P4 attenuates cyst breakdown and primordial follicle formation both *in vivo* and *in vitro*^11^,^12^. In bovine fetal ovaries where folliculogenesis occurs during gestation similar to that in human ovaries, P4 also inhibits primordial follicle formation; moreover, P4 levels in bovine fetal ovaries decrease immediately before primordial follicle formation^13^,^14^. Because of the importance of the quality and quantity of primordial follicles in fertility capacity, it can be suggested that high P4 levels in ovaries act as a physiological inhibitor of folliculogenesis to prevent oocytes from forming premature or abnormal follicles. The decrease in P4 levels after birth abolishes this suppressive effect, resulting in extensive follicle formation. Although Notch signaling pathway and tumor necrosis factor α are suggested to mediate the effect of P4 on follicle formation^9^,^15^, the exact mechanisms underlying this effect remain unclear.

Like other steroid hormones, P4 exerts its effects by specifically binding to its receptors, which include classic nuclear P4 receptors (nPRs), and putative membrane receptors of P4: members of the P4 and adipoQ receptor (PAQR) family membrane P4 receptor α (mPRα), mPRβ and mPRγ, and members of the membrane-associated P4 receptor family progesterone receptor membrane component 1 (PGRMC1) and PGRMC2^16^. Among them, PGRMC1 is a single-transmembrane protein containing a transmembrane N-terminal domain and an intracellular C-terminal cytoplasmic cytochrome b5 domain^17^, and specifically binds to P4 with high affinity^18^. PGRMC1 mediates the non-genomic effects of P4 on various physiological events such as preventing the apoptosis and proliferation of granulosa/luteal cells, regulating antral follicle development, and inhibiting gonadotropin-releasing hormone (GnRH) neuron activity^19^,^20^. PGRMC1 is also highly expressed in rat perinatal ovaries, in contrast, nPRs have low expression^15^. Moreover, missense mutations of PGRMC1 lead to premature ovarian failure (POF) in humans^21^. However, limited information is available on the role of PGRMC1 in early ovary development.

In the present study, we used an *in vitro* fetal ovary culture system to investigate whether PGRMC1 mediated P4-induced inhibition of primordial folliculogenesis and whether this effect involved the modulation of oocyte meiotic progression. We also examined whether cyclic AMP (cAMP), a vital messenger molecule that controls oogenesis, was involved in this process.

## Results

### P4–BSA inhibited primordial follicle formation

**Figure 1:**
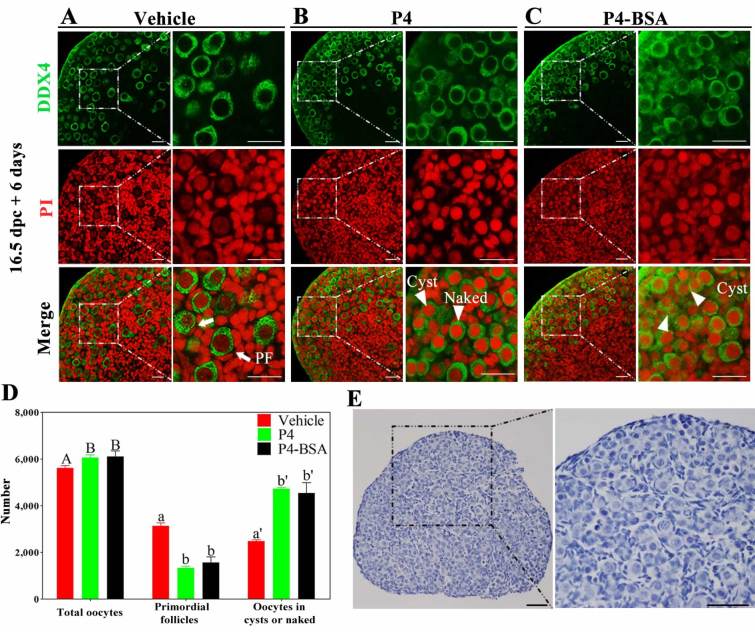
P4–BSA significantly inhibited primordial follicle formation *in vitro*. Ovaries at 16.5 dpc were cultured with vehicle (**A**), 1 μM progesterone (P4) (**B**), or 1 μM BSA-conjugated P4 (P4–BSA) (**C**) for 6 days. Paraffinized sections of the ovaries were obtained and were labeled with anti-DEAD box polypeptide 4 (DDX4) antibody (green) and propidium iodide (PI, red) for shape analysis (**A**–**C**). The numbers of oocytes and follicles were counted after staining the sections with hematoxylin (**D**). Arrowheads indicate unassembled oocytes: oocytes in cysts or naked oocytes, and arrows indicate primordial follicles; scale bar = 25 μm. Different letters denote statistical significance at *P* < 0.05 (total oocytes) and *P* < 0.001 (primordial follicles and oocytes in cysts or naked oocytes) (ANOVA and post hoc test, n = 6, 3 independent replicates). Then the paraffinized sections of 19.5 dpc ovaries were subjected to immunohistochemical analysis by staining with anti-nPRs antibody (**E**); scale bar = 25 μm.

To determine whether membrane P4 receptor or nPRs mediated the effect of P4 on primordial follicle formation, we used BSA-conjugated P4 (P4–BSA), a cell-impermeable form of P4 that does not bind to nPRs. For this, 16.5 dpc ovaries were cultured *in vitro* with vehicle, 1 μM P4, or 1 μM P4–BSA for 6 days. P4 treatment inhibited cyst breakdown and primordial follicle formation (Fig. 1B), which was consistent with that reported in previous studies^11^,^12^. Surprisingly, majority of oocytes in ovaries in the P4–BSA group remained in the cysts or were naked and only few assembled follicles were observed (Fig. 1C), which was similar to that observed in ovaries in the P4 group. The numbers of assembled follicles in ovaries in the P4 (1333 ± 44, 22.0% ± 0.7%) and P4–BSA (1572 ± 136, 25.7% ± 2.2%) groups were less than half of those in ovaries in the vehicle group (3138 ± 73, 55.9% ± 1.3%; Fig. 1D). To rule out the possibility that the effect of P4–BSA was due to free P4 decomposition, we compared the dose-dependent effects of P4 and P4–BSA. After treating 16.5 dpc ovaries with vehicle, 0.01–1 μM P4, or 0.01–1 μM P4–BSA for 6 days, the proportions of primordial follicles were counted. P4 inhibited primordial follicle formation at a dose-dependent manner as reported previously^11^,^12^. P4-BSA also displayed a dose-dependent repression of follicle formation, and the effects of P4 and P4-BSA were comparable at the same concentration (Supplementary Fig. S1). In addition, microarray analysis of transcriptome showed that nPRs were not expressed at detectable levels in 17.5 dpc and 3 dpp ovaries (Supplementary Table S1). And no immunohistochemical staining of nPRs was detected in 19.5 dpc ovaries (Fig. 1E), whereas the specific staining of nPRs was present in the positive tissue controls (Supplementary Fig. S2A,B). Thus, it is likely that membrane P4 receptors may mediate the effects of P4 on primordial follicle formation.

### PGRMC1 was highly expressed in oocytes in mouse prenatal ovaries

**Figure 2:**
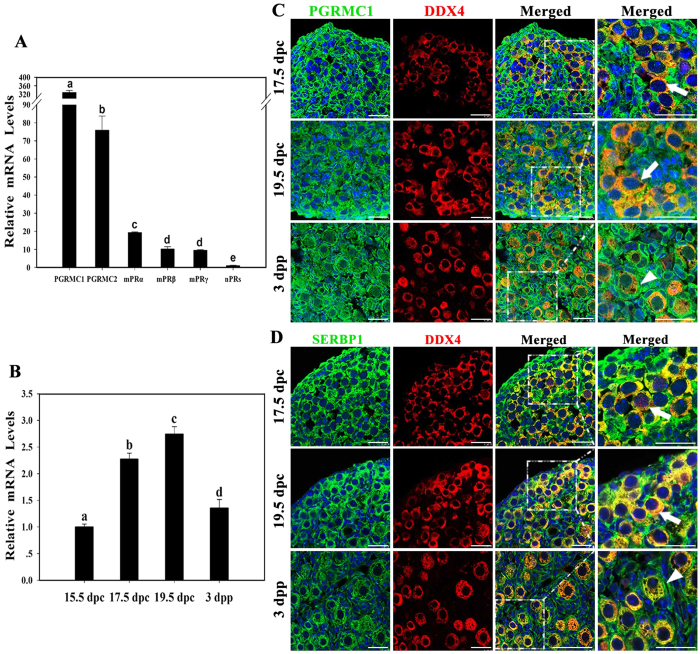
PGRMC1 was highly expressed in oocytes in mouse prenatal ovaries. The mRNA levels of membrane or nuclear P4 receptors in 17.5 dpc ovaries were assessed by performing qRT-PCR. Different letters denote statistical significance at *P* < 0.001 (ANOVA and post hoc test, 3 independent replicates, in each replicate every treatment contained 10 ovaries) (**A**). *Pgrmc1* mRNA levels in a mouse ovary obtained from 15.5 dpc to 3 dpp were measured by performing qRT-PCR. Different letters denote statistical significance at *P* < 0.005 (ANOVA and post hoc test, 3 independent replicates, in each replicate every treatment contained 10 ovaries) (**B**). Ovaries from 17.5 dpc to 3 dpp were collected and subjected to immunofluorescence analysis by labeling with anti-PGRMC1 (green) and anti-DDX4 (red) antibodies (**C**). In addition, the ovaries were immunostained with anti-SERBP1 (green) and anti-DDX4 (red) antibodies at the same time (**D**). Both PGRMC1 and SERBP1 were strongly expressed in oocytes before birth and were strongly expressed in pre-granulosa cells surrounding the oocytes after birth. Arrows indicate labeled oocytes, and arrowheads indicate labeled pre-granulosa cells; scale bar = 25 μm.

To further determine the P4 receptor involved in primordial follicle formation, mRNA levels of different P4 receptors in 17.5 dpc ovaries were examined by performing qRT-PCR. *Pgrmc1* was the most abundantly expressed P4 receptor in mouse fetal ovaries (Fig. 2A). The mRNA level of *Pgrmc1* was approximately 4 times that of *Pgrmc2* and >10 times that of *mPRα*, *mPRβ*, and *mPRγ*. In contrast, the mRNA levels of nPRs were very low.

Next, expression profile of *Pgrmc1* during primordial folliculogenesis was determined by performing qRT-PCR. *Pgrmc1* mRNA levels increased significantly from 15.5 dpc to 19.5 dpp and decreased after birth (Fig. 2B). Immunofluorescence analysis was performed to determine the cellular colocalization of germline marker DEAD box polypeptide 4 (DDX4, also known as MVH) and PGRMC1 or its partner serpine1 mRNA-binding protein 1 (SERBP1, also known as PAIRBP1 or RDA288). Strong immunofluorescence staining was observed for both PGRMC1 and SERBP1 in oocytes before birth and in pre-granulosa cells surrounding the oocytes after birth (Fig. 2C,D). No immunofluorescence was observed in control ovary sections (Supplementary Fig. S3A). The expression pattern of PGRMC1 suggested that PGRMC1 was involved in primordial follicle formation.

### Loss of function of PGRMC1 reversed P4-induced suppression of primordial folliculogenesis

**Figure 3:**
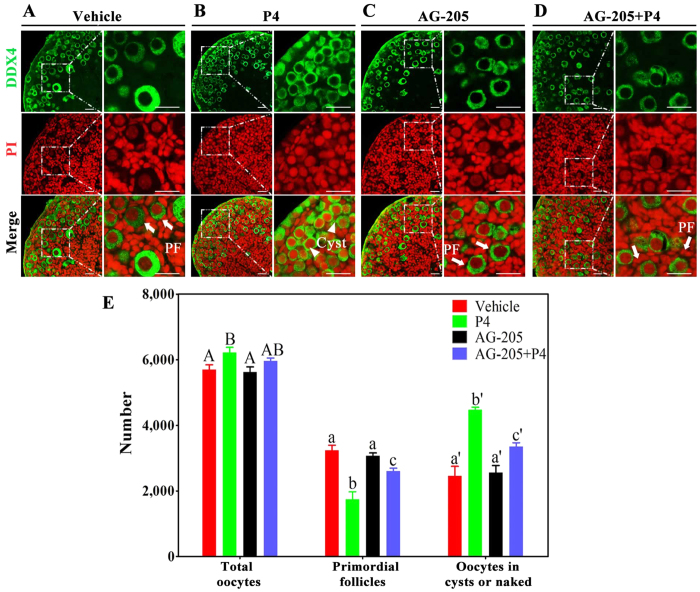
PGRMC1 inhibitor AG-205 reversed the suppressive effect of P4 on primordial follicle formation *in vitro.* For this, 16.5 dpc ovaries were isolated and were cultured with vehicle (**A**), 1 μM P4 alone (**B**), 5 μM AG-205 (PGRMC1 inhibitor) alone (**C**), or both AG-205 and P4 (**D**) for 6 days. Paraffinized sections of the ovaries were stained with anti-DDX4 (green) antibody and PI (red) for morphology analysis (**A**–**D**). Then the numbers of oocytes and follicles were counted after staining the sections with hematoxylin (**E**). Arrowheads indicate oocytes in cysts or naked oocytes, and arrows indicate primordial follicles; scale bar = 25 μm. Different letters denote statistical significance at *P* < 0.01 (ANOVA and post hoc test, n = 3–5, 3 independent replicates).

To determine whether PGRMC1 mediated the inhibitory effect of P4 on primordial follicle formation, we cultured 16.5 dpc ovaries with vehicle, P4, AG-205 (PGRMC1 inhibitor), or AG-205 plus P4 for 6 days. We observed that 57.0% ± 2.0% (3241 ± 78) oocytes in ovaries in the vehicle group and 54.6% ± 1.6% (3070 ± 53) oocytes in ovaries in the AG-205 group were assembled into primordial follicles (Fig. 3A,C,E). Ovarian sections in the P4 group showed markedly reduced cyst breakdown, with only 28.0% ± 1.7% (1747 ± 131) oocytes forming primordial follicles (Fig. 3B,E). Whereas in ovaries in the AG-205 plus P4 group, 43.8% ± 1.5% (2605 ± 90) oocytes broke apart from the cysts and formed primordial follicles (Fig. 3D,E).

**Figure 4:**
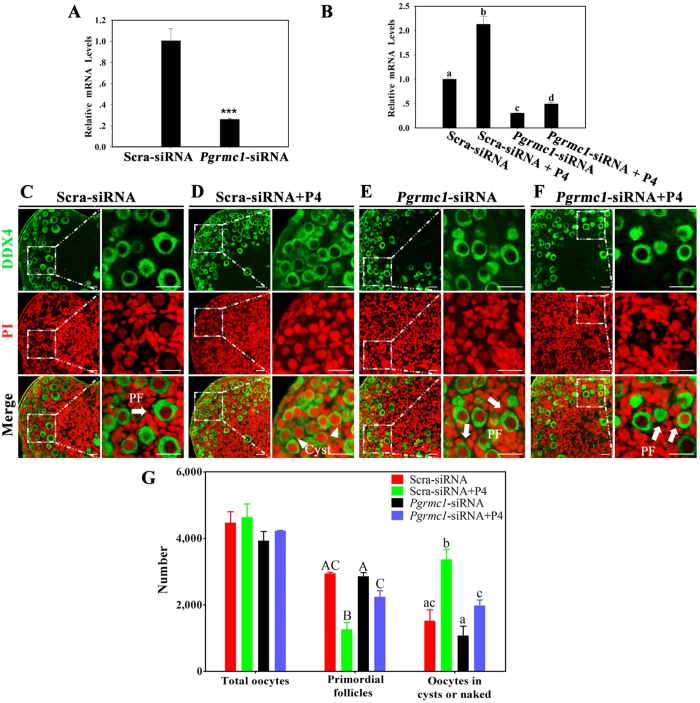
Downregulation of PGRMC1 by RNAi reversed P4-induced inhibition of primordial follicle formation *in vitro.* For this, 16.5 dpc ovaries were transfected with scrambled siRNA (Scra-siRNA) or siRNA against *Pgrmc1 (Pgrmc1*-siRNA) for 4 days, and transfection efficiency was determined by performing qRT-PCR (**A**); ***denotes statistical significance at *P* < 0.001 between vehicle and treated ovaries (*t*-test, 3 independent replicates, in each replicate every treatment contained 10 ovaries). Next, Scra-siRNA- or *Pgrmc1*-siRNA-transfected ovaries were treated with vehicle or P4 at the same time, and *Pgrmc1* expression was measured by performing qRT-PCR after 4 days of culture (**B**). Different letters denote statistical significance at *P* < 0.005 (ANOVA and post hoc test, 3 independent replicates, in each replicate every treatment contained 10 ovaries). After culturing for 6 days, the ovaries were fixed and embedded in paraffin, and the paraffinized sections were stained with anti-DDX4 antibody (green) and PI (red) for morphology analysis (**C**–**F**). Then the numbers of oocytes and follicles were counted after staining the sections with hematoxylin (**G**). Arrowheads indicate oocytes in cysts or naked oocytes, and arrows indicate primordial follicles; scale bar = 25 μm. Different letters denote statistical significance at *P* < 0.05 (ANOVA and post hoc test, n = 3–5, 3 independent replicates).

To further confirm the function of PGRMC1 in follicle formation, we knocked down *Pgrmc1* by performing RNA interference (RNAi). After 4 days of transfection, *Pgrmc1* mRNA levels in *Pgrmc1* siRNA-transfected ovaries decreased to 26% of those in scrambled siRNA-transfected ovaries (Fig. 4A). The efficiency of RNAi was also determined after exposing the ovaries to P4. Treatment with scrambled siRNA plus P4 significantly increased *Pgrmc1* expression relative to that after treatment with scrambled siRNA alone. However, *Pgrmc1* mRNA levels in *Pgrmc1* siRNA plus P4-treated ovaries were only 23% of those in scrambled siRNA plus P4-treated ovaries (Fig. 4B).

The shape and number of oocytes were determined after treating 16.5 dpc ovaries with scrambled siRNA, *Pgrmc1* siRNA, scrambled siRNA plus P4, or *Pgrmc1* siRNA plus P4 for 6 days. In ovaries in the scrambled siRNA plus P4 group, only 27.3% ± 2.6% (1267 ± 120) oocytes formed primordial follicles (Fig. 4D,G). In contrast, majority of oocytes (53.1% ± 4.3%, 2245 ± 182) in ovaries in the *Pgrmc1* siRNA plus P4 group dissociated from the cysts and assembled into primordial follicles (Fig. 4F,G). Additionally, 65.9% ± 0.4% (2948 ± 19) and 72.7% ± 1.6% (2860 ± 63) oocytes assembled into primordial follicles in ovaries in the scrambled siRNA and *Pgrmc1* siRNA groups, respectively (Fig. 4C,E,G). These results indicated that PGRMC1 mediated P4-induced suppression of primordial follicle formation.

### P4–PGRMC1 interaction repressed oocyte meiotic progression and pre-granulosa cell proliferation

**Figure 5:**
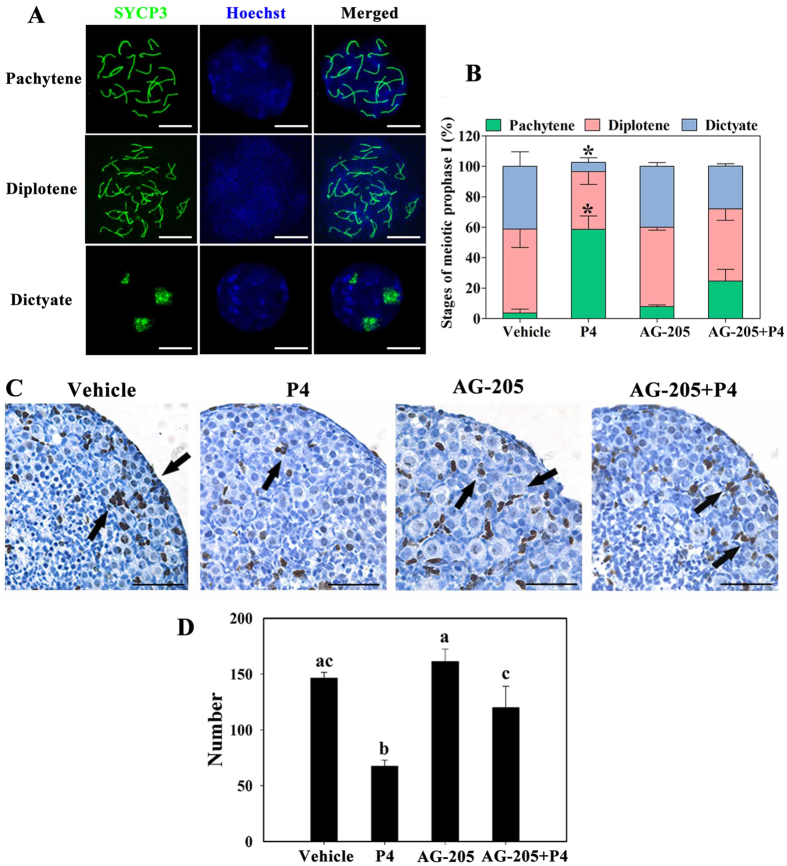
P4–PGRMC1 interaction suppressed meiotic progression and pre-granulosa cell proliferation. For this, 16.5 dpc ovaries were isolated and cultured with vehicle, 1 μM P4 alone, 5 μM AG-205 alone, or both AG-205 and P4 for 4 days. The spread chromosomes of cultured ovaries were labeled with anti-SYCP3 antibody (green) and Hoechst 33342 (blue) to identify (**A**) and analyze (**B**) the proportion of oocytes in each stage of meiotic prophase I; scale bar = 10 μm. *Denotes statistical significance at *P* < 0.05 between vehicle and treated ovaries (ANOVA and post hoc test, 3 independent replicates, in each replicate every treatment contained 3 ovaries). Next, the paraffinized ovary sections were subjected to immunohistochemical analysis by staining with anti-Ki67 antibody to determine cell proliferation (**C**). Arrows indicate proliferating pre-granulosa cells; scale bar = 25 μm. The number of Ki67-positive pre-granulosa cells was counted in the largest cross-section of each ovary from each treatment group (**D**). Different letters denote statistical significance at *P* < 0.05 (ANOVA and post hoc test, n = 3, 3 independent replicates).

Given that appropriate progression of the meiotic prophase I of oocytes is essential for primordial follicle formation, we determined whether P4–PGRMC1 interaction was involved in the meiotic progression of oocytes by performing synaptonemal complex protein 3 (SYCP3) staining in oocyte spread chromosomes. We observed that in vehicle-treated 16.5 dpc ovaries cultured for 4 days, more than half of the oocytes (55.1% ± 12.1%) were in the diplotene stage, 41.2% ± 9.7% oocytes progressed to the dictyate stage, and only 3.7% ± 2.6% oocytes remained in the pachytene stage. Meiotic distribution of oocytes in AG-205-treated ovaries was comparable to that in vehicle-treated ovaries. In contrast, in P4-treated ovaries, majority of oocytes (58.7% ± 8.8%) remained in the pachytene stage and only 6.1% ± 3.0% oocytes progressed to the dictyate stage. Addition of AG-205 plus P4 significantly relieved the meiotic delay observed after P4 treatment, with 24.6% ± 7.7% oocytes being in the pachytene stage, 47.4% ± 7.4% oocytes being in the diplotene stage, and 28.1% ± 1.6% oocytes being in the dictyate stage (Fig. 5A,B).

We also assessed somatic cell proliferation by performing immunohistochemical staining of cell proliferation marker Ki67. After a 4-day culture of 16.5 dpc ovaries, results of immunostaining showed that Ki67 was widely expressed in pre-granulosa cells surrounding the cysts in vehicle- (146.3 ± 3.0), AG-205- (161.3 ± 6.4), and AG-205 plus P4-treated (120.0 ± 11.0) ovaries. However, the number of stained pre-granulosa cells in the P4 group (67.3 ± 3.2) was approximately 50% of that in the other groups (Fig. 5C,D). Overall, P4–PGRMC1 interaction repressed meiotic progression and pre-granulosa cell proliferation in perinatal ovaries, suggesting that this interaction inhibited primordial follicle formation by suppressing the meiotic progression of oocytes and proliferation of pre-granulosa cells.

### P4–PGRMC1 interaction decreased cAMP levels

**Figure 6:**
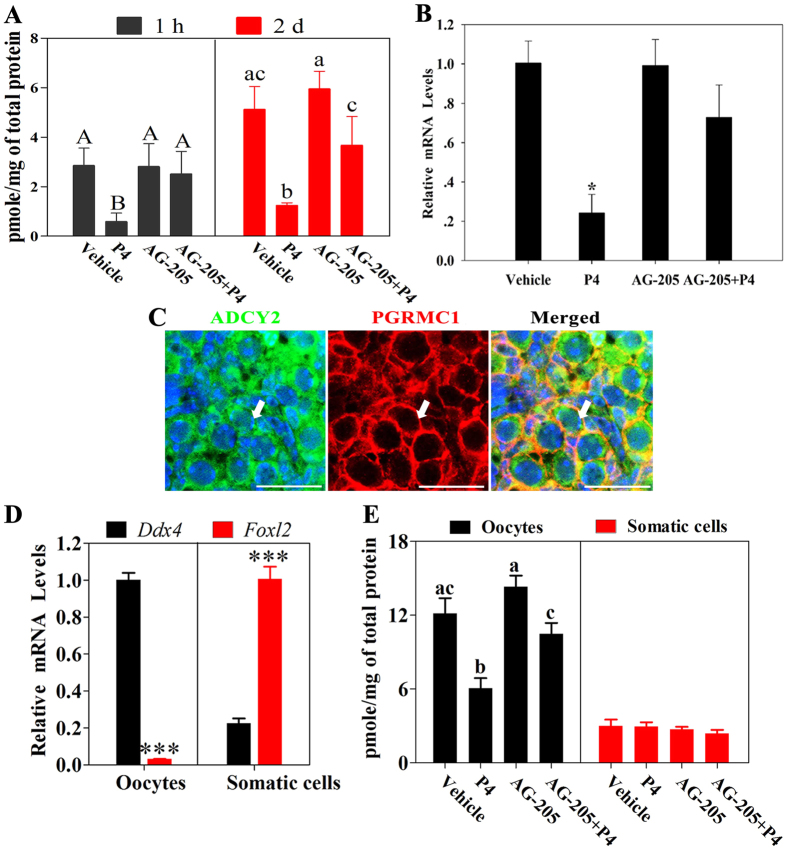
P4–PGRMC1 interaction decreased intra-oocyte cAMP levels. For this, 16.5 dpc ovaries were isolated and cultured with vehicle, 1 μM P4 alone, 5 μM AG-205 alone, or both AG-205 and P4. After 1 h or 2 days of culture, cAMP levels in the ovaries were measured by performing radioimmunoassay (RIA) (**A**). Different letters denote statistical significance at *P* < 0.05 (ANOVA and post hoc test, 3 independent replicates, in each replicate every treatment contained 30 ovaries). After 2 days of culture, *Adcy2* mRNA levels in ovaries in all the groups were analyzed by performing qRT-PCR (**B**). *Denotes statistical significance at *P* < 0.05 between vehicle and treated ovaries (ANOVA and post hoc test, 3 independent replicates, in each replicate every treatment contained 10 ovaries). Next, the ovaries at 19.5 dpc were subjected to immunofluorescence analysis by staining with anti-ADCY2 antibody (green) and anti-PGRMC1 antibody (red) (**C**). Results of immunofluorescence analysis showed that ADCY2 and PGRMC1 were predominately colocalized in the cytoplasm of oocytes. Arrows indicate labeled oocytes; scale bar = 25 μm. Then, oocytes and somatic cells were purified from fetal ovaries, and their expression of oocyte marker *Ddx4* and somatic cell marker *Foxl2* was measured by qRT-PCR (**D**). ***Denotes statistical significance at *P* < 0.001 (*t*-test, 3 independent replicates, in each replicate every treatment contained 10 ovaries). 16.5 dpc ovaries were cultured with vehicle, 1 μM P4 alone, 5 μM AG-205 alone, or both AG-205 and P4 for 2 days, and then oocytes and somatic cells were purified and performed RIA (**E**). Different letters denote statistical significance at *P* < 0.05 (ANOVA and post hoc test, 3 independent replicates, in each replicate every treatment contained 20-30 ovaries).

Our recent study showed that cAMP enhanced early meiotic progression and follicle formation^7^. Therefore, to investigate whether cAMP was regulated by P4–PGRMC1 interaction during early ovary development, we measured cAMP levels by performing radioimmunoassay (RIA) after culturing 16.5 dpc ovaries for 1 h or 2 days. Levels of cAMP decreased sharply after P4 treatment for 1 h or 2 days, compared with that after vehicle, AG-205, or AG-205 plus P4 treatment (Fig. 6A). Next, the expression of the cAMP-synthesizing enzyme in perinatal ovaries adenylyl cyclases 2 (*Adcy2*)^7^ was determined by performing qRT-PCR after culturing 16.5 dpc ovaries for 2 days. *Adcy2* mRNA levels decreased significantly after P4 treatment. In contrast, no significant difference was observed in *Adcy2* mRNA levels in vehicle-, AG-205-, or AG-205 plus P4-treated ovaries (Fig. 6B). Then, ADCY2 and PGRMC1 were stained by immunofluorescence in 19.5 dpc ovaries, in which PGRMC1 and ADCY2 were primarily colocalized in oocytes (Fig. 6C); and no fluorescence was observed in control ovary sections (Supplementary Fig. S3B). To further confirm in which cell type cAMP was modulated by P4, oocytes and somatic cells were purified after culturing 16.5 dpc ovaries for 2 days and the cAMP levels were measured by RIA. As shown in Fig. 6E, cAMP levels in oocytes declined significantly after P4 treatment relative to that after vehicle, AG-205, or AG-205 plus P4 treatment. However, cAMP levels in somatic cells showed no obvious changes after these treatments. These results suggested that cAMP in oocytes acted as a downstream component of the P4–PGRMC1 pathway during early ovary development.

### Dibutyryl cAMP abolished the effect of P4–PGRMC1 interaction on meiotic progression and folliculogenesis

**Figure 7:**
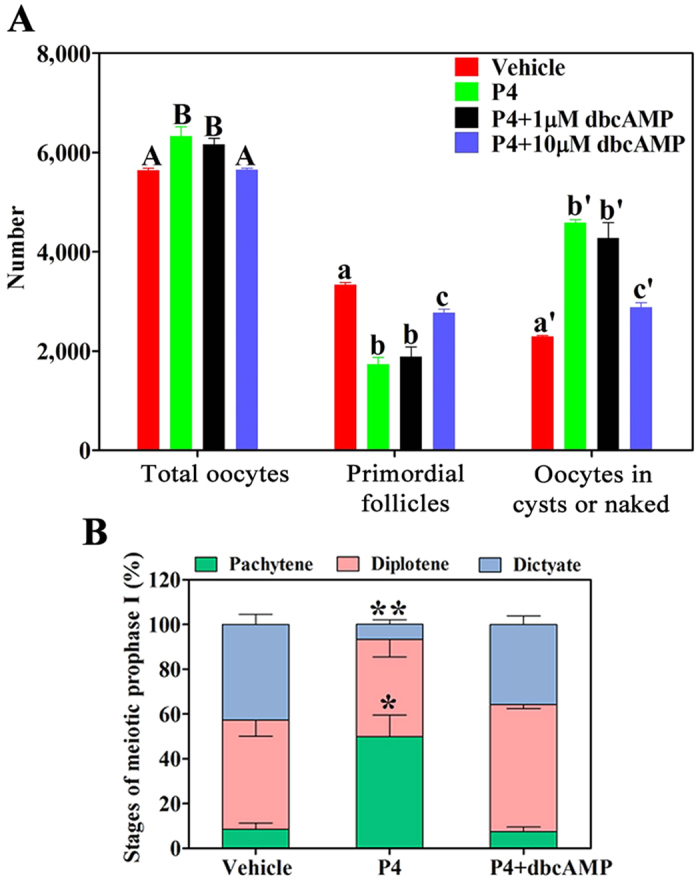
DbcAMP abolished the effect of P4–PGRMC1 on meiotic progression and folliculogenesis *in vitro.* For this, 16.5 dpc ovaries were cultured with vehicle, 1 μM P4 alone, 1 μM P4 plus 1 μM dibutyryl cAMP (dbcAMP, a cAMP analog), or 1 μM P4 plus 10 μM dbcAMP for 6 days. Paraffinized ovary sections were labeled with hematoxylin to determine the numbers of oocytes and primordial follicles (**A**). Different letters denote statistical significance at *P* < 0.05 (total oocytes) and *P* < 0.005 (primordial follicles and oocytes in cysts or naked oocytes) (ANOVA and post hoc test, n = 3–5, 3 independent replicates). Next, 16.5-dpc ovaries were cultured with vehicle, 1 μM P4 alone, or 1 μM P4 plus 10 μM dbcAMP for 4 days. SYCP3 staining of spread chromosomes was performed to analyze the proportion of oocytes in each stage of meiotic prophase I (**B**). *Denotes statistical significance at *P* < 0.05, and **denotes statistical significance at *P* < 0.005 between vehicle and treated ovaries (ANOVA and post hoc test, 3 independent replicates, in each replicate every treatment contained 3 ovaries).

To verify whether cAMP mediated the effect of P4-PGRMC1 on meiosis progression and folliculogenesis, a cell permeable cAMP analog dibutyryl-cAMP (dbcAMP) was employed. After a 6-day culture of 16.5 dpc ovaries, we observed that approximately 59.3% ± 0.7% (3342 ± 38) oocytes were enclosed by pre-granulosa cells in vehicle-treated ovaries. In contrast, only 27.5% ± 2.1% (1738 ± 134) and 30.6% ± 3.1% (1888 ± 193) oocytes in P4- and 1 μM dbcAMP plus P4-treated ovaries, respectively, assembled into follicles. Addition of 10 μM dbcAMP abolished P4-induced inhibition of primordial follicle formation, with 49.0% ± 1.3% (2771 ± 74) oocytes being assembled into primordial follicles (Fig. 7A).

Meiotic progression was also examined after culturing 16.5 dpc ovaries for 4 days. Approximately half of the oocytes (49.9% ± 9.7%) remained in the pachytene stage and 6.8% ± 2.1% oocytes progressed to the dictyate stage in P4-treated ovaries. In contrast, only 7.6% ± 2.1% oocytes remained in the pachytene stage and 35.8% ± 3.8% oocytes progressed to the dictyate stage in 10 μM dbcAMP plus P4-treated ovaries, which was similar to that observed in vehicle–treated ovaries (Fig. 7B). Together, these results indicated that P4–PGRMC1 interaction inhibited oocyte meiotic progression and primordial follicle formation by decreasing cAMP levels.

### mPRα was regulated by PGRMC1 during primordial folliculogenesis

**Figure 8:**
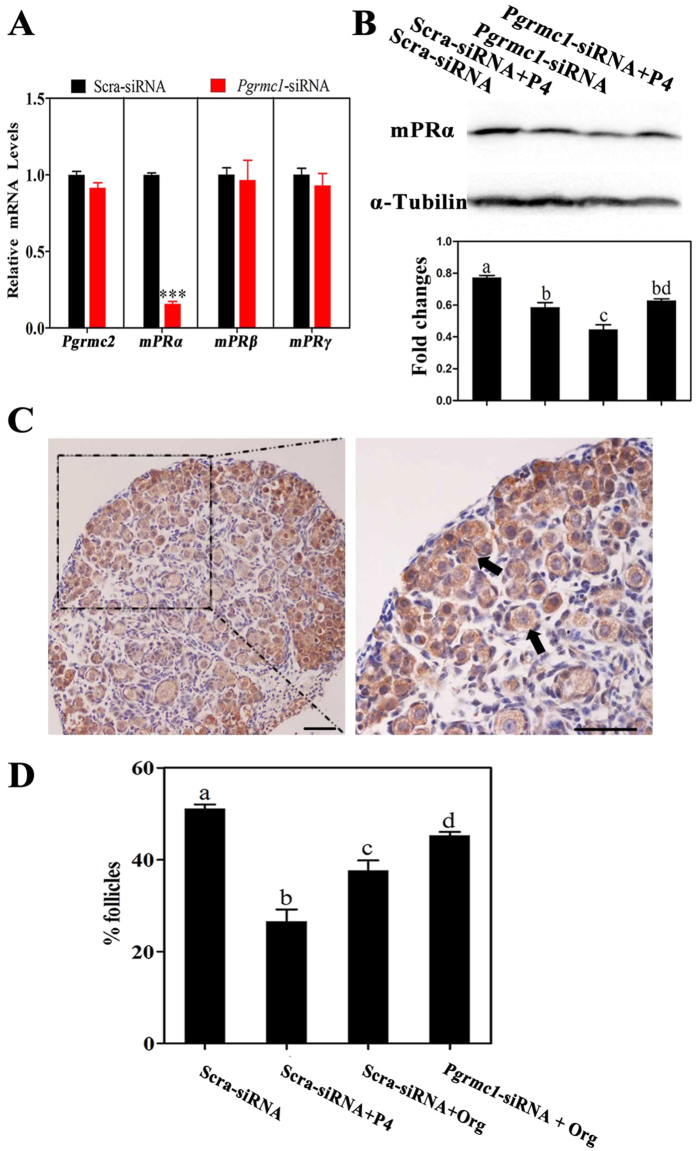
mPRα was regulated by PGRMC1 during primordial folliculogenesis. For this, 16.5 dpc ovaries were transfected with scrambled siRNA (Scra-siRNA) or siRNA against *Pgrmc1 (Pgrmc1*-siRNA) for 4 days, and the expression of *Pgrmc2*, *mPRα,mPRβ* and *mPRγ* was determined by performing qRT-PCR (**A**); ***denotes statistical significance at *P* < 0.001 between vehicle and treated ovaries (*t*-test, 3 independent replicates, in each replicate every treatment contained 10 ovaries). Next, Scra-siRNA- or *Pgrmc1*-siRNA-transfected ovaries were treated with vehicle or P4 at the same time, after 4 days of culture cell membrane protein was extracted and mPRα expression was measured by performing Western blot (**B**). Different letters denote statistical significance at *P* < 0.05 (ANOVA and post hoc test, 3 independent replicates, in each replicate every treatment contained 15 ovaries). Ovaries from 19.5 dpc were collected and subjected to immunohistochemical analysis by labeling with anti-mPRα antibodies (**C**). Arrows indicate labeled oocytes; scale bar = 25 μm. 16.5 dpc ovaries were treated with scrambled siRNA, scrambled siRNA plus 1 μM P4, scrambled siRNA plus 1 μM mPRα-specific agonist Org OD 02-0 (Org), or *Pgrmc1* siRNA plus 1 μM Org for 6 days. Paraffinized ovary sections were labeled with hematoxylin to determine the percentage of primordial follicles (**D**). Different letters denote statistical significance at *P* < 0.05 (ANOVA and post hoc test, n = 3–5, 3 independent replicates).

Recently, PGRMC1 and mPRα were reported to mediate similar effects of P4 on some target cells^22^,^23^,^24^. And PGRMC1 could directly interact with G protein-coupled receptors (GPCRs) including mPRα, and modulate their expression, location or function, in a P4-dependent manner^24^,^25^. Therefore, we investigated whether mPRα was regulated by PGRMC1 here, and whether mPRα was also involved in primordial follicle formation. In brief, 16.5 dpc ovaries were treated with scrambled siRNA or *Pgrmc1* siRNA for 4 days. After that, qRT-PCR results showed that *mPRα* mRNA levels in *Pgrmc1* siRNA-transfected ovaries decreased significantly compared with those in scrambled siRNA-transfected ovaries, whereas *Pgrmc2*, *mPRβ* and *mPRγ* mRNA levels had no marked differences (Fig. 8A). Then, we measured the plasma membrane protein levels of mPRα by Western blot. Treatment with *Pgrmc1* siRNA also significantly decreased mPRα levels, relative to that after treatment with scrambled siRNA (Fig. 8B). By immunohistochemistry, mPRα was moderately expressed in oocytes of 19.5 dpc ovaries (Fig. 8C).

Furthermore, 16.5 dpc ovaries were treated with scrambled siRNA, scrambled siRNA plus 1 μM P4, scrambled siRNA plus 1 μM mPRα-specific agonist Org OD 02-0 (Org), or *Pgrmc1* siRNA plus 1 μM Org for 6 days. The results showed that 37.6% ± 2.2% oocytes formed follicles in the scrambled siRNA plus Org group, which were significantly less than that in the scrambled siRNA group (51.4% ± 0.9%), but more than that in the scrambled siRNA plus P4 group (27.8% ± 2.6%). And knockdown of PGRMC1 partially abolished the suppression of Org in follicle formation (45.8% ± 0.7%) (Fig. 8D). Overall, our results implied that the function of mPRα was regulated by PGRMC1 in early ovary development.

## Discussion

Previous studies have indicated that P4 inhibits primordial follicle formation^11^,^12^,^13^,^14^. In our study, P4–BSA exerted a similar effect to that of P4 on follicle formation and nPRs were not expressed in perinatal ovaries, indicating the involvement of a membrane P4 receptor. PGRMC1 was highly expressed in oocytes before birth, and loss of function of PGRMC1 abolished P4-induced suppression of follicle formation, meiotic progression, and pre-granulosa cell proliferation. Interestingly, P4–PGRMC1 interaction decreased intra-oocyte cAMP levels, and dbcAMP abolished the effects of P4 on meiotic prophase I and primordial follicle formation. Overall, these results indicated that P4–PGRMC1 interaction attenuated oocyte meiotic progression and primordial follicle formation by decreasing intra-oocyte cAMP levels.

Non-classical P4 receptors might be responsible for P4 action in primordial follicle formation. The classical P4 receptors nPRs, as ligand-activated, nuclear transcription factors, mediate many critical physiological functions of P4, such as establishment and maintenance of pregnancy^26^. However, disorganization of primordial follicle pool does not occur in nPRs-null mice^27^. Moreover, nPRs antagonist mifepristone (RU486) cannot abolish P4-induced inhibition of primordial follicle formation^9^,^11^. In the current study, qRT-PCR showed that mRNA levels of nPRs were the lowest (less than one-hundred of the mRNA levels of *Pgrmc1*) among all P4 receptors in 17.5 dpc ovaries. And microarray analysis indicated that mRNA levels of nPRs were undetectable in mouse perinatal ovaries, which was consistent with the previous microarray report in rat^15^. More important, no specific staining of nPRs was observable in perinatal ovaries. P4–BSA, which cannot enter cells to activate nPRs, inhibited primordial follicle formation to a similar extent as that by P4. Therefore, we concluded that nPRs were not expressed in perinatal ovaries and that a membrane P4 receptor may mediate the effects of P4 on primordial folliculogenesis.

PGRMC1 has been proved to be important for mammals’ gonad development. In recent years, P4 is found to elicit a variety of fast, non-genomic effects on many physiological events, which are mediated by membrane P4 receptors, including PGRMC1 and PGRMC2^28^ and mPRα, mPRβ as well mPRγ^29^. Missense mutations or reduced expression of PGRMC1 leads to premature ovarian failure (POF) in humans, which may be induced by the impaired binding of cytochrome P450 7A1 to PGRMC1^21^,^30^,^31^. Clinical investigations suggest that PGRMC1 is involved in early ovary development similar to other candidate genes associated with POF^32^, such as genes encoding forkhead box l2 (FOXL2)^33^, newborn ovary homeobox (NOBOX)^34^, and factor in germline alpha (FIGLA)^35^. In the present study, PGRMC1 was primarily studied because PGRMC1 and its partner SERBP1 were the most highly expressed genes during primordial follicle formation, as determined by microarray analysis^15^ and qRT-PCR. We have proved that PGRMC1 transcripts maintained at high levels before parturition and then dropped after birth, which was consistent with the changes in ovarian P4 levels reported previously^8^,^10^. In addition, loss of function of PGRMC1 abolished P4-induced inhibition of cyst breakdown and follicle formation. Therefore, PGRMC1 mediated the effects of P4 on primordial follicle formation in this study.

Considering the suppressive function of P4-PGRMC1 interaction in folliculogenesis, it is critical to discover the mechanisms behind this action. Accelerated or disordered progression of oocyte meiotic prophase I increases or disrupts primordial follicle assembly^6^,^7^,^36^,^37^,^38^, suggesting that appropriate progression of oocyte meiotic prophase I is required for primordial folliculogenesis. Results of the present study showed that P4–PGRMC1 interaction controlled the progression of meiotic prophase I in oocytes, which was consistent with the effects of P4 and PGRMC1 on the late stage of oocyte meiosis. P4 participates in gonadotropin-induced meiosis resumption of mammalian oocytes^39^, which is probably independent of nPRs^27^. Moreover, PGRMC1 is expressed on the centromeres of chromosomes after meiosis resumption, and antibody treatment results in chromosomal disorganization in bovine oocytes^40^. In the present study, the proliferation of pre-granulosa cells surrounding the cysts, which contribute to primordial follicle formation^41^, was also inhibited by P4–PGRMC1 interaction. Collectively, these data suggested that P4–PGRMC1 interaction attenuated primordial follicle formation by blocking the progression of oocyte meiotic prophase I and proliferation of pre-granulosa cells.

P4-PGRMC1 interaction may relate to homeostasis of cAMP levels within oocytes before parturition. It is well-known that in adult ovaries a high level of cAMP within immature oocytes maintains the arrest of meiotic prophase I. Our recent study also shows that cAMP contributes to oocyte meiotic prophase I progression and primordial folliculogenesis. And cAMP levels gradually increase accompanied by meiosis progression in perinatal ovaries, and attain peak levels when oocytes are arrested at the dictyate stage^7^. Results of the present study showed that P4–PGRMC1 interaction decreased cAMP levels in oocytes and that dbcAMP abolished P4–PGRMC1 interaction-induced inhibition of oocyte meiotic prophase I and follicle formation, indicating that the effect of P4–PGRMC1 interaction was mediated by a decrease in intra-oocyte cAMP levels. As long as majority of oocytes enter diplotene and dictyate stages after birth^7^, which coincides with the sharp decline in ovarian P4 and PGRMC1 levels and the increase of intra-oocyte cAMP levels, we speculate that before birth, endogenous P4–PGRMC1 interaction maintains moderate levels of cAMP in oocytes^7^, to likely ensure accuracy of chromosome synapsis as well as fine-tuned meiosis progression^42^. After birth, cAMP levels increase and improve follicle formation and meiosis progression, until oocytes are arrested in the dictyate stage. This may be one of the reasons for explaining accelerated meiotic progression accompanied by rapid primordial follicle formation in *in vitro* cultured fetal ovaries.

However, P4–PGRMC1 interaction may not regulate intra-oocyte cAMP levels directly, because there are no reports on the direct interaction between PGRMC1 and cAMP so far. On the other hand, increasing studies indicate that PGRMC1 interacts with GPCRs, directly^25^,^43^. For example, PGRMC1 can bind with mPRα but not mPRβ or mPRγ, to modulate mPRα location and activity in cell line models^24^. And PGRMC1 and mPRα mediate similar effects of P4 on antiapoptotic actions in granulosa and cancer cells, as well as on GLP-1R insulinotropic actions in beta cells^24^,^25^. Consistent with these reports, the current study implies that mPRα and PGRMC1 were both expressed in oocytes. And the expression and effects of mPRα were regulated by PGRMC1. Altogether, mPRα was regulated by PGRMC1, and may be partially involved in primordial follicle formation. Thus, we speculate that the action of PGRMC1 in regulating primordial follicle formation and intra-oocyte cAMP levels may be partially dependent on mPRα. However, the interacting patterns between PGRMC1 and mPRα in early ovary development seems still elusive, and needs further investigations.

In conclusion, we showed that PGRMC1 as a membrane P4 receptor, mediated the inhibitory effects of P4 on oocyte meiotic progression and primordial folliculogenesis by decreasing intra-oocyte cAMP levels. These observations increase our understanding of molecular endocrine mechanisms underlying primordial folliculogenesis and may help elucidate mechanisms underlying the pathogenesis of human diseases associated with follicle insufficiency.

## Methods

### Animals

All the experiments and animal procedures were conducted in accordance with the Guidelines of the Animal Experiments and Experimental Animals Management Committee of Capital Medical University. The study protocol was approved by the Animal Experiments and Experimental Animal Welfare Committee of Capital Medical University (Permit Number: 2013-X-023). ICR mice used in this study were purchased from Beijing Vital Laboratory Animal Technology Co. (Beijing, China). Eight-week-old adult mice were mated overnight to induce pregnancy. The morning after mating (when a vaginal plug was observed) was designated as 0.5 dpc.

### *In vitro* fetal ovary culture

16.5 dpc ICR mouse ovaries were isolated, as described previously^44^. Dissected ovaries were cultured in 24-well culture dishes (Nunclon, Nunc, Roskilde, Denmark) containing 1 mL DMEM/F-12 medium (Gibco, Gaithersburg, MD, USA) supplemented with the chemicals at 37 °C and in 5% CO_2_. The chemicals used in the study were P4 (0.01–1 μM), dbcAMP (1–10 μM), AG-205 (5 μM; Sigma-Aldrich, St. Louis, MO, USA), Org OD 02-0 (1 μM; Axon Medchem, Groningen, Netherland), and P4–BSA, which was backwashed with ether to ensure that no free P4 was present; and extremes of heat might make some of the compound decompose, but the amide link would withstand in most situations (0.01–1 μM; AbD Serotec, Raleigh, NC, USA).

### Immunohistochemical and immunofluorescence staining

Ovaries were fixed in 4% polyformaldehyde (PFA) at 4 °C, embedded in paraffin, and cut into 5-μm-thick sections. After dewaxing, rehydration, antigen retrieval, and serum blocking, whole ovary sections were immunostained, as described previously^45^. For analyzing morphology and localization by performing immunofluorescence staining, the sections were incubated overnight with primary antibodies at 4 °C, followed by incubation with Alexa Fluor 488- or Alexa Fluor 555-conjugated secondary antibodies (Invitrogen Corporation, Carlsbad, CA, USA) for 1 h at 37 °C. All the primary antibodies and their dilutions are presented in Supplementary Table S2. The sections were stained with Hoechst 33342 or propidium iodide (PI; Sigma-Aldrich) for 5 min. Fluorescence images were obtained using TCS SP5 downright microscope (Leica, Wetzlar, Germany).

For assessing cell proliferation or localization by performing immunohistochemical staining, the ovary sections were incubated overnight with primary antibodies at 4 °C, followed by incubation with biotinylated secondary antibodies (Zhongshan, Beijing, China) for 15 min at room temperature and ABC complex (Zhongshan) for 15 min at room temperature. Peroxidase activity was detected using 3,3′-diaminobenzidine (Zhongshan). The sections were counterstained with hematoxylin, dehydrated, coverslipped with histomount reagent, and imaged using BMI4000B downright microscope (Leica). The number of proliferating pre-granulosa cells was counted in 2 serial sections of the largest cross-section of each ovary and was averaged, as described previously^46^.

### Counting the numbers of oocytes and follicles

The numbers of oocytes and follicles were counted using a generally accepted approach. Briefly, the ovaries were serially sectioned into 5-μm-thick sections, and the sections were placed in order on microscope slides. After hematoxylin staining, every fifth section was counted for the presence of oocytes and follicles. Cumulatively, oocyte and follicle numbers for each ovary were multiplied by 5.

### Quantitative RT-PCR

Total RNA was isolated using TRIzol Reagent (Thermo Fisher Scientific, San Jose, CA, USA). Next, 1 μg of the total RNA from each group was reverse transcribed using oligo(dT) primers and Moloney murine leukemia virus reverse transcriptase (Promega, Madison, WI, USA), according to the manufacturer’s instructions. Quantitative RT-PCR (qRT-PCR) was conducted to quantify mRNA levels of target genes by using IQ5 Real-time PCR Detection System (Bio-Rad, Hercules, CA, USA) and QuantiTect^®^ SYBR Green PCR kits (Qiagen, Valencia, CA, USA). In each experiment, relative fold changes in the expression of the selected genes were normalized to a specific group by using 2^−ΔΔCt^ method. All primer sequences used for performing qRT-PCR are listed in Supplementary Table S3.

### RNAi in fetal ovaries

RNAi in cultured fetal mouse ovaries was performed as described previously^9^. Briefly, 0.5 μL 20 μM siRNA (GenePharma, Shanghai, China) was injected into 16.5 dpc ovaries by using glass pipettes under a stereomicroscope. Next, the ovaries were electrotransfected by applying three 5-ms-long quasi-square pulses at pulse-field strength of up to 40 V/cm. The sequence of *Pgrmc1* siRNA, which targeted 895–913 bp of *Pgrmc1* mRNA, was 5′-GCTGTAATGCAAATGGTAAdTdT-3′ and that of the scrambled siRNA, which showed no significant homology to any known mouse mRNAs, was 5′-TTCTCCGAACGTGTCACGdTdT-3′.

### Chromosome spreads and immunofluorescence staining

Ovaries were manipulated as described in our previous study^47^. Briefly, the ovaries were digested with 0.25% trypsin solution at 37 °C. After centrifugation, the trypsinized ovaries were suspended and fixed in 1% PFA and were dried on glass slides. The ovary sections were then incubated overnight with anti-SYCP3 antibody (dilution, 1:100) at 37 °C, followed by incubation with Alexa Fluor 488-conjugated secondary antibodies for 1 h at 37 °C and 5 μg/mL Hoechst 33342 for 5 min. SYCP3 staining was performed to identify chromosomal axial elements during meiotic prophase I. Zygotene, pachytene, diplotene, and dictyate stages of meiotic prophase I were distinguished based on the appearance of axial elements^48^,^49^. In all, 300 oocytes from 2–3 ovaries were imaged for each slide and were counted using TCS SP8 STED downright microscope (Leica).

### Isolation of oocytes and somatic cells

Oocytes and somatic cells in fetal ovaries were isolated as described previously^50^. Briefly, after washing in PBS, ten 17.5–19.5 dpc ovaries were digested in 0.25% trypsin solution for 5 min at 37 °C and then for 30 min at 4 °C. The trypsinized ovaries were pipetted up and down, till no lumps were observed. The reaction was terminated by adding fetal bovine serum (FBS). After centrifugation, the precipitated cells was resuspended by PBS and filtered through 20 μm diameter cytoscreener. The filtered cells were harvested by centrifuging.

### Measurement of cAMP levels

In all, ovaries or isolated cells from each treatment group were ground carefully and were dissolved in 100 μL 0.1 M HCl. After resting on ice for 10 min, the solutions were snap-frozen in liquid nitrogen and were stored at −80 °C. For RIA, the samples were rethawed and were centrifuged at 12,000 × g for 10 min. Supernatants were collected and were dried overnight at 60 °C. Next, cAMP levels were measured by performing RIA with cAMP [^125^I] RIA kit (IZOTOP, Budapest, Hungary), according to the manufacturer’s instruction. The protein concentration was measured by the BCA protein assay kit (Applygen, Beijing, China).

### Western blot

Membrane proteins were extracted with *ProteinExt*^TM^ Mammalian Membrane Protein Extraction Kit (Transgen Biotech, Beijing, China). Aliquots of proteins were resolved by 10% SDS–PAGE and transferred to pieces of Protran nitrocellulose membrane (Schleicher & Schuell, Dassel, Germany). The membranes were blocked with 5% nonfat dry milk for 1 h and incubated overnight at 4 °C with primary antibodies. After five washes in TBST, the membranes were incubated with HRP-conjugated secondary antibody (1:5000, Zhong Shan) for 1 h at room temperature. The proteins on the membranes were visualized using the SuperSignal chemiluminescent detection system (Thermo Fisher Scientific). The density of blot was measured by AlphaEaseFC Software if needed.

### Statistical analysis

Statistical analysis was performing using *t*-test or ANOVA with StatView software (SAS Institute, Cary, NC, USA). When a significant *F* ratio was defined by ANOVA, data were further analyzed using Fisher’s protected least significant difference post hoc test. *P* < 0.05 was considered statistically significant. For each experiment, each treatment group included 3–30 perinatal ovaries; moreover, each experiment was repeated at least 3 times. Values are expressed as mean ± standard error of the mean.

## Additional Information

**How to cite this article**: Guo, M. *et al.* Progesterone Receptor Membrane Component 1 Mediates Progesterone-Induced Suppression of Oocyte Meiotic Prophase I and Primordial Folliculogenesis. *Sci. Rep.* **6**, 36869; doi: 10.1038/srep36869 (2016).

**Publisher’s note:** Springer Nature remains neutral with regard to jurisdictional claims in published maps and institutional affiliations.

## Supplementary Material

Supplementary Information
