# Supplementary material for: Progesterone Receptor Membrane Component 1 Mediates Progesterone-Induced Suppression of Oocyte Meiotic Prophase I and Primordial Folliculogenesis
Source: Sci Rep. 2016 Nov 16;6:36869. doi: 10.1038/srep36869 (PMC5111101; doi:10.1038/srep36869)
Supplement: Supplementary Information [file srep36869-s1.pdf]

Supplemental Data

Progesterone Receptor Membrane Component 1 Mediates Progesterone-Induced Suppression of Oocyte Meiotic Prophase I and Primordial Folliculogenesis

Meng Guo[1], Cheng Zhang[2], Yan Wang[3], Lizhao Feng[4], Zhengpin Wang[5], Wanbo Niu[4], Xiaoyan Du[1], Wang Tang[3], Yuna Li[3], Chao Wang[4], Zhenwen Chen[3]

Figure S1.

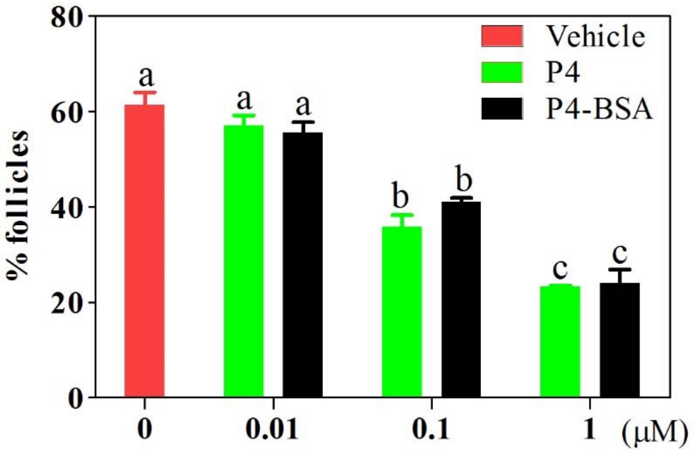

Figure S1. Comparison of the dose-dependent effects of P4 and P4-BSA on primordial follicle formation. Ovaries at 16.5 dpc were cultured with vehicle, 0.01-1 μM P4, or 0.01-1 μM P4-BSA for 6 days, respectively. Then paraffinized ovary sections were labeled with hematoxylin to determine the percentage of primordial follicles. Different letters denote statistical significance at $P < 0.05$ (ANOVA and post hoc test, n = 3-5, 3 independent replicates).

Figure S2.

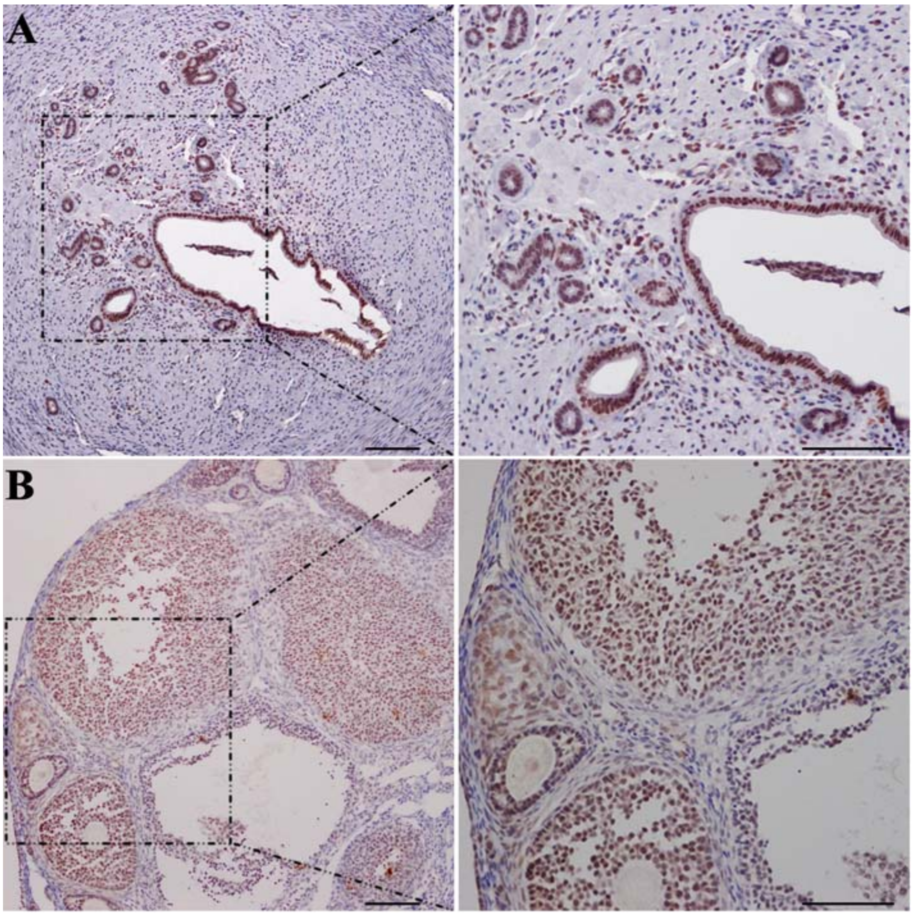

Figure S2. The positive tissue controls of immunohistochemistry localization of nPRs. 4 dpc maternal uterus (A) and adult ovaries (B) in mice were immunostained with anti-nPRs antibody under the same experimental conditions with Fig. 1E. nPRs were strongly stained in endometrium cells in uterus, and moderately stained in granulosa cells and luteal cells in adult ovaries; scale bar = 50 μm.

Figure S3.

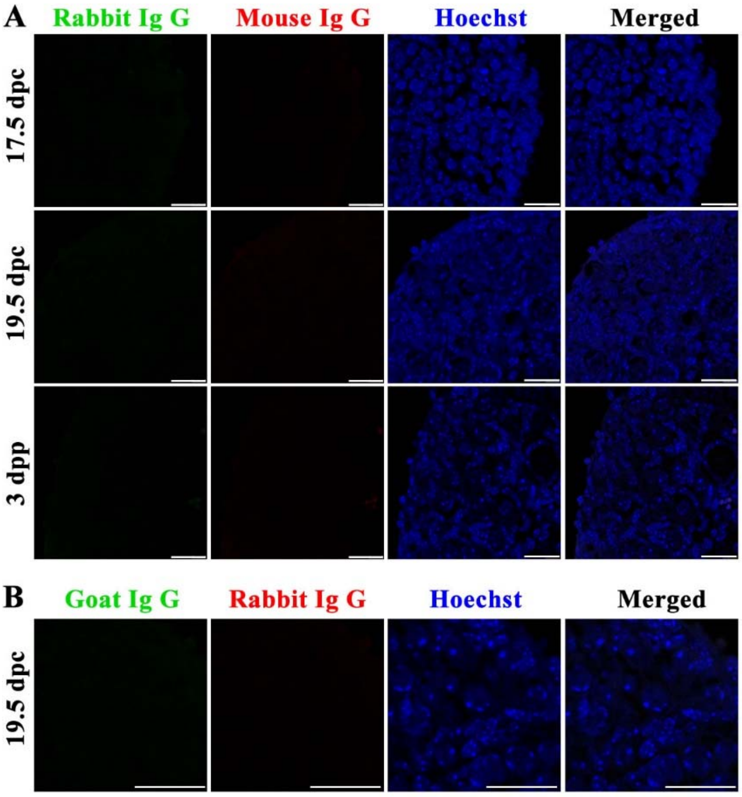

Figure S3. The negative controls of immunofluorescence colocalization using isotype-matched IgG. Ovaries were immunostained with the isotype-matched IgG of anti-PGRMC1 and anti-SERBP1 antibodies (green), and with the isotype-matched IgG of anti-DDX4 antibody (red) under the same experimental conditions with Fig. 2C-D (A). Ovaries were immunostained with the isotype-matched IgG of anti-ADCY2 antibody (green) and the isotype-matched IgG of anti-PGRMC1 antibody (red) under the same experimental conditions with Fig. 6C (B); scale bar = 25 μm.

Figure S4.

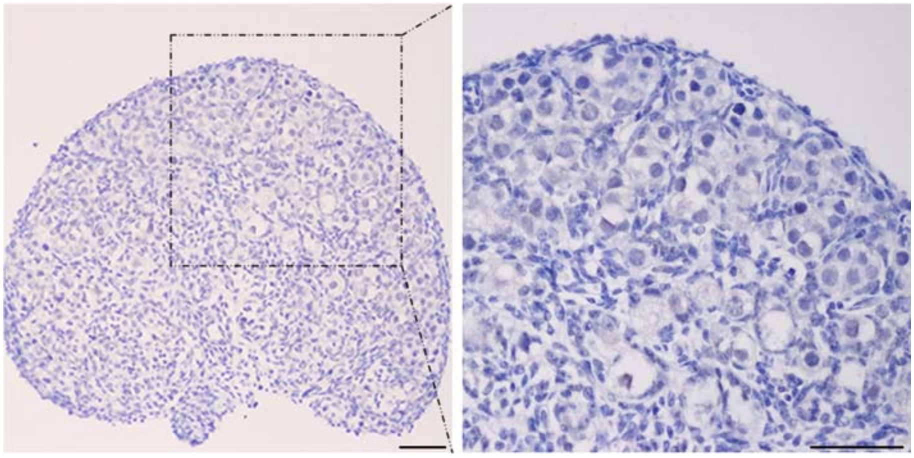

Figure S4. The negative controls of immunohistochemistry localization of mPRα using isotype-matched IgG. 19.5 dpc ovaries were immunostained with the isotype-matched IgG of anti- mPRα antibody under the same experimental conditions with Fig. 8C; scale bar = 25 μm.

Table S1. Microarray results for *Pgrmc1*, *nPRs* and several other essential genes in mouse perinatal ovaries.

Gene	Description	17.5 dpc		3 dpp	
		Signal	Detection	Signal	Detection
Ddx4	DEAD box polypeptide 4	3831.3	P	2362.2	P
Figla	factor in germline alpha	6816.4	P	5208.5	P
Zp3	zona pellucida glycoprotein 3	95.1	P	10687.1	P
Nobox	newborn ovary homeobox	1926.1	P	4849.5	P
Gata4	GATA binding protein 4	324.1	P	552.3	P
Gata6	GATA binding protein 6	2619	P	1977.7	P
Wt1	wilms tumor 1	1572.4	P	1684.4	P
Sry	sex determining region of Chr Y	13.1	A	17.5	A
Pgrmc1	**progesterone receptor membrane component 1**	**6499.3**	**P**	**6791**	**P**
nPRs	**nuclear progesterone receptor A and B**	**2.7**	**A**	**4.3**	**A**

The microarray results referred from a published doctorate dissertation of our laboratory, *The research on the mechanisms of mouse primordial folliculogenesis* by Zhang, H., China Agricultural University, 2008, P63-73 (In Chinese). Gene expression was measured with an Affymetrix (Affymetrix, Santa Clara, CA, USA) Mouse 430 v2.0 expression array, including 45000 probes, 34000 mouse genes.

Table S2. Primary antibodies used in our study.

Antibody	Source	Cat. number	Dilution		
			IF	IHC	WB
DDX4	Abcam	ab27591	1:200		
PGRMC1	Sigma-Aldrich	HPA002877	1:200		1:1000
SERBP1	Abcam	ab55993	1:200		
SYCP3	Novus	NB300-232	1:100		
Ki67	CST	12202s		1:400	
ADCY2	Santa Cruz	sc-32112	1:100		
nPRs	DAKO	A0098		1:100	
mPRα	Abcam	ab75508		1:200	1:750
α-tubulin	CST	2125			1:1000

IF, immunofluorescence; IHC, immunohistochemistry; WB, Western blot.

Table S3. Primers used in our study.

Genes	Forwards (5’-……..-3’)	Backwards (5’-……..-3’)
Pgrmc1	CCATCAACGGCAAGGTGTTC	GCAGGGGTGAGGTCAGAAAG
Pgrmc2	TGGGAAAGTCTTCGACGTGAC	GTGCATCCTTATCCAGGCAGA
nPRs	CTCCGGGACCGAACAGAGT	ACAACAACCCTTTGGTAGCAG
mPRα	CGTGTTGCACCGCATCATAG	TCCCTGCCCAAAGATGTGAC
mPRβ	TACCAGGGACGCCATGAGAT	CCTCAGCCCGTAATACATATTAA
mPRγ	TACCAGGGACGCCATGAGAT	CCTCAGCCCGTAATACATATTAA
Adcy2	GATCTGCTTTCCAAGCCGAAG	GCATGTATTGACGCTCGGGT
Ddx4	GCTTCATCAGATATTGGCGAT	GCTTGGAAAACCCTCTGCTT
Foxl2	AGAGGCTCACTCTGTCCGGC	TCTGCCAGCCCTTCTTGTTCT
Gapdh	GGTGAAGGTCGGTGTGAACG	CTCGCTCCTGGAAGATGGTG
